# An Account of the Great Efficacy of the Digitalis Purpurea in Dropsies

**Published:** 1785

**Authors:** 


VII. *An Account of the great Efficacy of the Digitalis Purpurea in Dropſies. Communicated in a Letter to Dr.* Simmons, *by* John Warren, M. D. *Phyſician at Taunton in Somerſetſhire.*

AS I feel myſelf much intereſted for the fate of the Digitalis purpurea, of which ſome notice has been lately taken in the London Medical Journal *, and fear, from the repreſentation there made, that the ſame deſtiny may await it, which the Peruvian bark formerly met with, I am deſirous of communicating to you ſome general account of the effects which I have experienced from its adminiſtration; and

* See page 55.

which, I truſt, may prove a means of ſubſtantiating its reputation, and of placing it in the liſt of the highly valuable remedies afforded us by the vegetable world.

I was firſt induced to make a trial of the virtues of this medicine about ſix years ago, in conſequence of having received a well-authenticated report of an hydropic patient being perfectly cured by it, under the care of ſome empiric, when he had been judged irremediable by the attending practitioners.

The firſt perſon to whom I gave it, was a gentleman rather advanced in life, a man of temperance, but whoſe conſtitution had of late been greatly broken down, through the prevalence of ſcorbutic acrimony, and a ſedentary mode of living. He had laboured under anaſarca and hydrothorax for ſome months, and an aſcites was now ſupervening very rapidly. He had rarely paſſed, for many preceding weeks, more than half a pint of urine (always of the deepeſt red colour) in the courſe of twenty-four hours, notwithſtanding his thirſt obliged him to take at leaſt ſix times that quantity of fluids in the ſame ſpace. Some briſk cathartics of jalap and cryſtals of tartar, elaterium, gamboge, &c. had, from time to time, been given him through through the progrefs of his difeafe, but with little apparent advantage; and whenever taken, the languor confequent on the operation of thefe purgatives, was fo great, as at length to determine him to make no farther trial of their ufe. Squill medicines in various forms, with alkaline falts, and the ufual remedies commonly recommended in fimilar cafes, were likewife ufed with as little benefit. His ftrength was now totally deftroyed, and the fwellings and difficulty of refpiration were increafed to that degree, as to incapacitate him for all kinds of motion; in fhort, his fituation appeared to himfelf, as well as to all his friends, perfectly defperate. Such were the circumftances, when I directed for him the following decoction:

℞. Folior. Digital. (flore) purpur. recent. unc. iij. Coque ex aquæ fontanæ ℔. ifs ad unc. ix. et cola. Colaturæ addendo aquæ Junip. c. Syr. e Cort. Aurant. āā unc. ifs fiat Miftura.

Of this it was recommended him to take two table fpoonfuls every four hours, until it induced vomiting, purging, or a copious flow of urine. The fecond day after commencing this plan, a great degree of naufea enfued, which was foon followed by frequent efforts to vomit; of urine then took place, and in the courſe of about thirty-ſix hours he diſcharged nearly ſeven gallons of water, and was perfectly cured, without any other aſſiſtance than what was afforded by exerciſe and a generous dietetic plan.

Another caſe occurred to me not long after, which likewiſe placed the efficacy of this medicine in a conſpicuous point of view, although the termination of the diſeaſe for which it was given proved fatal. This patient was alſo advanced in life, and temperate; had laboured under anaſarca and aſcites for many months; voided but a ſmall quantity of urine, and of a deep red colour; and had taken nearly the ſame medicines above ſpecified, and with as little advantage. His abdomen, legs, and thighs, were enlarged to a moſt enormous ſize, and he was in every reſpect incapable of the ſlighteſt exertion. The decoction of the Fox Glove was adminiſtered in the morning, and repeated every four hours. About nine o'clock the following night, it began its operation, by vomiting, purging, and a vaſt flow of urine; but through the great inattention and neglect of thoſe attendant on him, no cordial, nor any kind of ſupport whatever was given through the courſe of of its violently operating for ten hours; when the poor man, thoroughly evacuated, ſunk entirely through exhauſtion; and a life was ſacrificed by want of care, which might, in all probability, have otherwiſe been preſerved, and at this time borne ample teſtimony to the ineſtimable value of the remedy now under conſideration.

The third caſe in which I made trial of the Digitalis purpurea, was that of a lady about thirty-ſix years of age, whoſe diſeaſe appeared to me to be a dropſy of the right ovarium; ſhe had been gradually enlarging in the right epigaſtrium for ſeveral months; had paſſed very little urine for a long ſeaſon; was become much emaciated; and had a great deſpondency about her. She had, for many weeks previous to my ſeeing her, been taking the Pilulæ ſcilliticæ, and the Acetum ſcilliticum, in ſuch doſes as her ſtomach would bear, but without the leaſt advantage. The decoction, as above, was immediately directed, and produced its uſual evacuating effects, and ſhe was perfectly cured by it in the ſpace of four days. This happened five years ago; and the great number of dropſical patients I have ſince had, through this lady's kind recommendation, bear conſtant teſ-

timony timony of the gratitude ſhe feels for her deliverance from a malady, which ſhe is perſuaded would, but for this remedy, have proved fatal.

From this period, I have conſtantly employed the Fox Glove in all caſes of Hydrothorax, Aſcites, Anaſarca, and in Dropſies of the Ovaria, when I had reaſon to ſuſpect that they did not owe their origin to diſeaſed viſcera; and from long experience of its effects, I can in general tolerably well aſcertain, *a priori*, the caſes in which it will prove ſerviceable. Such appear to me to originate from a laxity of the exhalants depending on general debility, which is in fact that ſpecies of dropſy, the moſt frequent of any. This ſtate of the ſyſtem in its firſt appearance ſeems to be what has been conſidered as a particular diſeaſe under the name of Cachexy; but ought, as Dr. Cullen properly obſerves, to be regarded as the beginning of a general dropſy, and is what he ſtyles the *Hydropic Diatheſis*. But I do not mean to inſinuate, that I have found the Digitalis purpurea infallible in all ſuch caſes; for I muſt candidly confeſs, that even here it has frequently diſappointed my hopes: I only wiſh to be underſtood, derſtood, that when the *Hydropic Diatheſis* has previouſly prevailed, I have experienced from it infinitely more advantage, than from any other medicine; and that I have repeatedly cured my patients with it, when all other remedies have baffled my moſt ſanguine expectations. So diſſimilar is this medicine in its effects to the general claſs of anti-hydropics, which require a ſubſequent tonic courſe, that my admiration has been frequently excited by what means a permanent cure is effected by it, without the uſe of corroborants.

Phyſicians the leaſt converſant with practice, cannot fail of being well acquainted with the very uncertain operation of all kinds of diuretics; and though the kidneys appear to afford the moſt natural outlet for a great part of the watery fluids contained in the blood veſſels, &c. and the increaſing of the excretion thence to a conſiderable degree, is a means the moſt likely of any, to excite an abſorption in dropſical parts; yet as no medicine, that we are acquainted with, arranged under the claſs of diuretics, will in all caſes uniformly exert this, their ſpecific power, ſo it is no reflection on the Digitalis purpurea, (though the moſt certain tain of any) that it ſhares a portion of the ſame fallibility with the reſt of its claſs *.

Again, that ſucceſs cannot conſtantly be expected to await the exhibition of this medicine, is apparent, when we reflect that the remote cauſes of dropſy are very frequently certain diſeaſes previouſly exiſting in the animal œconomy, and which are to be cured by the remedies particularly adapted to theſe diſeaſes; the removing of which may be often difficult: but when ſuch remote cauſes cannot be counteracted, the cure of the dropſy muſt be difficult, and perhaps impoſſible.

Whether or not it be neceſſary to the operation of this medicine, that it ſhould previouſly exert its emetic powers, ere it can excite a copious flow of urine, I have not yet been able to aſcertain: but this I have conſtantly obſerved, that I never knew it to effect a cure,

* The late Mr. Darwin appears to have thought, from the caſes related by him, that where the urine is in due quantity, and of a natural (ſtraw) colour, the prognoſtic is favourable; but with reſpect to the latter, I have never been able to diſcover, that ſalutary effects were more readily produced by the Fox Glove, when one kind of urine was made, than another. —*Experiments eſtabliſhing a criterion between mucaginous and purulent matter.* Page 105.

nor to produce confiderable benefit, unlefs in cafes where it had at firft acted as an emetic. What makes it ftill more probable that its action on the ftomach is intimately connected with its producing falutary effects, may be inferred from the well-known obfervation, that *fpontaneous* vomiting has frequently excited an abforption in hydropic parts, and thereby drawn off the water lodged in them; and hence the practice of exciting a vomiting by art, which has been often employed with the greateft advantage. But if the emetic properties of the medicine be not neceffarily connected with its diuretic action, it would truly be a fortunate difcovery, could any mode of exhibiting it be found out, by which it might be divefted of this harraffing effect; for its vomiting powers are in general fo violent, and the kind of ficknefs which it induces fo peculiarly diftreffing, that I have frequently met with the greateft difficulty to prevail on my patients to have recourfe to it a fecond time, fhould the difeafe recur; notwithftanding their conviction of its highly falutary properties, and their readily confeffing they had before been folely indebted to it for their prefervation.

As far as a folitary fact can go, the following proves the Digitalis purpurea capable of curing this difeafe, without exerting any action on the ftomach. An ingenious apothecary of this town, who for fome years has treated his dropfical patients in the fame manner as myfelf, has fent me this cafe, which he thus relates:

"In the month of March laft, I was defired "to vifit a lad, about nine years of age, who "was dropfical to the greateft poffible degree. "His trunk was become three times its natural "fize, the fcrotum was nearly as big as his head, "which was likewife much enlarged, and his "extremities were fwollen in proportion. He "had been ill, and increafing in fize for two "months before I faw him; during which time "a variety of medicines had been given, but "without the leaft effect. I immediately di- "rected half an ounce of the decoctum mi- "rabile *, (for fo I muft call it) to be given him "twice daily. The two firft days little altera- "tion was difcovered; but on the third, he "began to bring off water in immenfe quan- "tities; for in forty-eight hours he made "twenty-feven pints, which were meafured;

* This was the *Decoct. Digital. purpur.* before fpecified.

and

Digitalis, efficacy of in Dropsies

" and his mother informed me, ſhe believed " many pints more had been paſſed during that " time, which could not be ſaved. The me- " dicine was continued ſome days longer, du- " ring which the urine was meaſured, until it " amounted in the whole to twenty-four quarts, " when the child appeared to be reduced to his " natural ſize; and he has perfectly recovered " his health and ſtrength. Had the boy taken " larger doſes, there is no doubt but it would " have operated much ſooner; for in every caſe " where I have given it, the deſired effect has " been very ſoon produced: I ſay, every caſe, " and I have adminiſtered it to great numbers " of both ſexes, and of all ages, not only in " anaſarca but aſcites, and have not failed in " any one of curing, or affording conſiderable " relief. I have only to remark, that this pa- " tient was not made ſick by the medicine; " which is the only inſtance of the kind that " has occurred in my practice, for in general " the ſickneſs produced by it is almoſt to " death."

I have been lately informed, that a Tincture of the Fox Glove, made by infuſing the root in proof ſpirit, has been found equally ſucceſs- ful with a decoction of the leaves; if ſo, I

Tincture of the Root, to be effectual

ſhould, *a priori*, be led to conceive, that the ſickneſs induced by the former would be much leſs than that occaſioned by the latter; for as in many reſpects it reſembles the ſquills in its action, which I find may be given in double their common doſe, and with nearly double advantage, if blended with ſome aromatic ſpirit, ſo this medicine may probably have its nauſeating properties in ſome degree counteracted, by being exhibited in an aromatic ſpirituous vehicle. Would not alſo an extract of it, joined with ſuch ſubſtances, prove equally ſerviceable?

An apothecary of my acquaintance in an adjacent town, to whom I not long ſince recommended a trial of this medicine, has lately informed me, that he had juſt before cured two hydropic patients by its leaves in ſubſtance: his mode of giving them was by blending about five grains powdered with ten grains of ſoap, and this quantity was adminiſtered twice or thrice daily, until the evacuating effects ſupervened. For my own part, I have hitherto been pretty conſtantly in the habit of uſing the green leaves, which can at all times be readily procured in the ſummer ſeaſon, and I have ſeldom met with any difficulty in collecting them, in this neighbourhood, during the winter. They are not then, then, it is true, fo fucculent, but a lefs quantity appears to anfwer every purpofe.

That the Digitalis purpurea fhould, according to the account of Dr. Karr, have failed of producing equally falutary purpofes in the North of Britain, which it has effected in the Weft, is, in my opinion, no fair argument againft it. In Scotland, the great degree of tone and vigour, induced by the rigour of the climate, cannot favour the hydropic diathefis. I am ftrengthened in this belief, from an obfervation I frequently made during the courfe of my ftudies at Edinburgh, refpecting dropfies, that they were in that country *morbi rariffime fanabiles*, and that in eight cafes out of ten, they commonly originated from great intemperance through dram-drinking; now and then from a check of perfpiration.

I think it right to obferve, that whenever I prefcribe the Fox Glove, I likewife order a warm cordial Julep to be fent with it, of which a few fpoonfuls are directed to be taken occafionally during the violence of its operation; but it has been my conftant practice never to perfift in the ufe of the decoction, if the expected flow of urine does not enfue in the courfe of of a few hours ſubſequent to its exerting its emetic or purgative qualities.

On the whole, it has been adminiſtered by me with ſo much ſucceſs, and ſo perfectly am I convinced of its ſuperior efficacy to every other medicine yet known for curing the dropſy, that I am perſuaded it needs only to have a general trial made of it, to be univerſally eſteemed in this diſeaſe that *optimum Dei donum* which I have found it. It is alſo my firm opinion, that if the knowledge of the effects of this valuable herb was alone confined to ſome empiric, as much reputation would attend it, and as large a property be amaſſed by it, as has reſulted to the proprietors of the Liege medicine for the gout, or of Dr. James's celebrated powder for fever.

If you judge the above remarks worthy of inſertion in the London Medical Journal, they are perfectly at your ſervice for that, or any other purpoſe.

*Taunton,*

May 2, 1785.

---

VIII. *Ac-*

